# Efficacy and Safety of a Novel Thrombectomy Device in Patients With Acute Ischemic Stroke: A Randomized Controlled Trial

**DOI:** 10.3389/fneur.2021.686253

**Published:** 2021-08-12

**Authors:** Yongxin Zhang, Weilong Hua, Zifu Li, Ya Peng, Zhian Han, Tong Li, Congguo Yin, Shoucun Wang, Guangxian Nan, Zhenwei Zhao, Hua Yang, Bin Zhou, Tianxiao Li, Yiling Cai, Jianmin Zhang, Guifu Li, Xiaoxiang Peng, Sheng Guan, Junshan Zhou, Ming Ye, Liqin Wang, Lei Zhang, Bo Hong, Yongwei Zhang, Jieqing Wan, Yang Wang, Qing Zhu, Jianmin Liu, Pengfei Yang

**Affiliations:** ^1^Department of Stroke Center, Changhai Hospital, Naval Military Medical University, Shanghai, China; ^2^Department of Neurosurgery, The First People's Hospital of Changzhou, Changzhou, China; ^3^Zhongshan City People's Hospital, Zhongshan, China; ^4^Department of Neurology, The Second Nanning People's Hospital, Nanning, China; ^5^Department of Neurology, Affiliated Hangzhou First People's Hospital, Zhejiang University School of Medical, Hangzhou, China; ^6^Department of Neurology, The First Hospital of Jilin University, Jilin, China; ^7^Department of Neurology, China-Japan Union Hospital of Jilin University, Jilin, China; ^8^Department of Neurosurgery, Tangdu Hospital the Fourth Military Medical University, Xi'an, China; ^9^Department of Neurosurgery, The Affiliated Hospital of Guizhou Medical University, Guizhou, China; ^10^The Fifth Affiliated Hospital of Sun Yat-Sen University, Zhuhai, China; ^11^Henan Provincial People's Hospital, Zhengzhou, China; ^12^PLA Strategic Support Force Characteristic Medical Center, Beijing, China; ^13^Department of Neurosurgery, The Second Affiliated Hospital of Zhejiang University School of Medicine, Hangzhou, China; ^14^Guangdong Provincial Hospital of Traditional Chinese Medicine, Guangzhou, China; ^15^Department of Neurology, The Third People's Hospital of Hubei Province, Wuhan, China; ^16^The First Affiliated Hospital of Zhengzhou University, Zhengzhou, China; ^17^Nanjing First Hospital, Nanjing Medical University, Nanjing, China; ^18^Renji Hospital Affiliated to Shanghai Jiaotong University School of Medicine, Shanghai, China; ^19^Medical Research & Biometrics Center, National Center for Cardiovascular Disease, China; ^20^Zhuhai Ton-Bridge Medical Tech. Co., Ltd., Zhuhai, China

**Keywords:** acute ischemic stroke, large vessel occlusion, thrombectomy, reperfusion, stent

## Abstract

**Purpose:** The Tonbridge stent is a novel retriever with several design improvements which aim to achieve promising flow reperfusion in the treatment of acute ischemic stroke (AIS). We conducted a randomized controlled, multicenter, non-inferiority trial to compare the safety and efficacy of the Tonbridge stent with the Solitaire FR.

**Methods:** AIS patients aged 18–85 years with large vessel occlusion in anterior circulation who could undergo puncture within 6 h of symptom onset were included. Randomization was performed on a 1:1 ratio to thrombectomy with either the Tonbridge stent or the Solitaire FR. The primary efficacy endpoint was successful reperfusion using a modified thrombolysis in cerebral infarction score (mTICI) of 2b/3. Safety outcomes were symptomatic intracranial hemorrhage (sICH) within 24 ± 6 h and all-cause mortality within 90 days. A clinically relevant non-inferiority margin of 12% was chosen as the acceptable difference between groups. Secondary endpoints included time from groin puncture to reperfusion, National Institutes of Health Stroke Scale (NIHSS) score at 24 h and at 7 days, and a modified Rankin Scale (mRS) score of 0–2 at 90 days.

**Results:** A total of 220 patients were enrolled; 104 patients underwent thrombectomy with the Tonbridge stent and 104 were treated with the Solitaire FR. In all test group patients, the Tonbridge was used as a single retriever without rescuing by other thrombectomy devices. Angioplasty with balloon and/or stent was performed in 26 patients in the Tonbridge group and 16 patients in the Solitaire group (*p* = 0.084). Before angioplasty, 86.5% of those in the Tonbridge group and 81.7% of those in the Solitaire group reached successful reperfusion (*p* = 0.343). Finally, more patients in the Tonbridge group achieved successful reperfusion (92.3 vs. 84.6%, 95% CI of difference value 0.9–16.7%, *p* < 0.0001). There were no significant differences on sICH within 24 ± 6 h between the two groups. All-cause mortality within 90 days was 13.5% in the Tonbridge group and 16.3% in the Solitaire group (*p* = 0.559). We noted no significant differences between groups on the NIHSS at either 24 h or 7 days and the mRS of 0–2 at 90 days.

**Conclusion:** The trial indicated that the Tonbridge stent was non-inferior to the Solitaire FR within 6 h of symptom onset in cases of large vessel occlusion stroke.

**Clinical Trial Registration:**ClinicalTrials.gov, number: NCT03210623.

## Introduction

Five landmark trials have established endovascular thrombectomy as one of the most powerful treatments for acute ischemic stroke (AIS) due to large vessel occlusion in anterior circulation (1–5). The benefits shown in these trials were driven by improved stent-retriever thrombectomy devices combined with patient selection. The Solitaire FR (Medtronic Inc., Irvine, CA, USA) has been one of the most frequently used stent retrievers. Recently, various novel thrombectomy devices have been developed and put into use.

**Figure 1:**
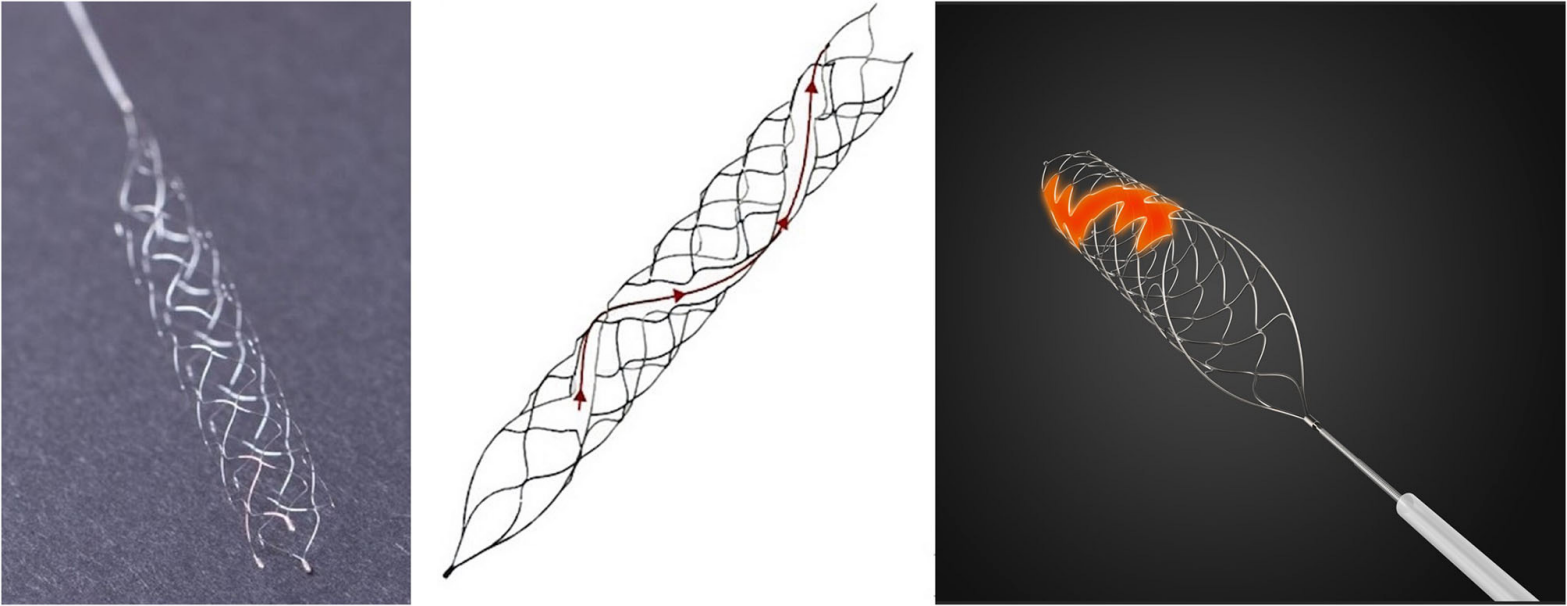
The Tonbridge device and the unique design of the longitudinal spiral opening along its tubular surface.

The Tonbridge stent (Tonbridge, Tonbridge Medical Technology, Zhuhai, Guangzhou, China) has been modified into a new design with a longitudinal spiral opening along its tubular surface (Figure 1). It has also been modified so that the finished temperature of nitinol increases the radial force, and has a broad size ranging from 3/4/5/6 mm. The maximum length of the series 4/5/6 mm is 30 mm. In *in vitro* tests, the Tonbridge stent had similar maximum friction within the 0.021-in. microcatheter and a slight increase in radial force when compared with the Solitaire FR. An *in vivo* comparative study in beagle models showed that the Tonbridge stent was safe and had a similar number of retriever attempts and similar recanalization rates when compared with the Solitaire FR (6). To evaluate the true efficacy and safety of this new device compared with that of the Solitaire FR in a clinical setting, a multicenter randomized controlled trial was designed and carried out.

## Methods

### Study Design and Patients

We conducted a randomized, prospective, controlled, multicenter, single-blind, non-inferiority trial with blinded outcome assessment that enrolled patients with AIS. This study was designed with input from an academic steering committee and overseen by an independent clinical events committee as well as an independent core laboratory. The sponsor of the study, Tonbridge Medical Technology, was responsible for the logistical operations and monitoring of the trial. An independent contract research organization (OSMUNDA Medical Device Service Group, en.osmundacn.com/) and a site management organization (Excellence Future International Consulting Co. Ltd., http://www.chncro.com) were also involved in monitoring the study in order to ensure the quality of the trial. All statistical analyses were conducted by four independent external statisticians (Medical Research & Biometrics Center, National Center for Cardiovascular Disease, China).

We enrolled patients from 17 tertiary care centers, which were each required to have performed at least 30 endovascular thrombectomy procedures during the previous year. The protocol was approved by the respective ethics committee of each participating site. Written informed consent was obtained from all patient participants or their legal representatives prior to enrollment. The trial was designed to enroll 220 patients with the following eligibility criteria: (1) adults aged 18–85 years of age; (2) a baseline National Institutes of Health Stroke Scale (NIHSS) score <30; (3) an angiographically proven occlusion in the internal carotid artery (ICA), middle cerebral artery (MCA) (M1 or M2), or the anterior cerebral artery (MCA) (A1 or A2); (4) a prestroke modified Rankin Scale (mRS) (7) score of <2; and (5) patients able to undergo puncture within 6 h of symptom onset. Key exclusion criteria were the following: a massive cerebral infarction (8, 9); an Alberta Stroke Program Early CT Score (ASPECTS) (10) of <6, or infarct volume ≥70 ml, or >1/3 of blood supplying areas on CT/diffusion-weighted imaging; simultaneous acute bilateral carotid occlusion; uncontrolled hypertension (defined as SBP >185 mmHg or DBP >110 mmHg after medication); concomitant use of oral anticoagulation medications; an INR >3.0; and a platelet count of <40 ×10^9^.

### Randomization and Blinding

Randomization was performed utilizing a 1:1 ratio to mechanical thrombectomy with either the Tonbridge stent or the Solitaire FR. This was accomplished by employing a web-based system with stratification according to the participating site. Treatment-group assignment was known to the operating physicians but blinded to the patients. Three postprocedure clinical follow-up exams were performed by independent physicians who were unaware of the treatment-group assignment of the patient.

### Procedures

According to guidelines, intravenous thrombolysis with rtPA was administered as a bridging therapy in patients who had no contraindications for it. The use of general anesthesia was performed according to standard practices based on local practice. A baseline angiogram was obtained before device deployment in order to assess angiographic inclusion and exclusion criteria. The choice of thrombectomy device was made according to random allocation. The instructions of the manufacturers regarding the Tonbridge stent were very similar to those of the Solitaire FR. Other retrievers, such as the Trevo stent (Stryker, Kalamazoo, MI, USA), or other techniques were allowed after three unsuccessful attempts with the Tonbridge stent. Aspiration with an intermediate catheter was allowed in both stent retriever arms. If there was an underlying stenosis or insufficient reperfusion, salvage measures, including additional balloon (Gateway, Boston Scientific, Natick, MA, USA) angioplasty and/or placement of a permanent stent (Enterprise, Johnson and Johnson, Raynham, MA, USA; Wingspan, Stryker, Kalamazoo, MI, USA) or an Apollo balloon-mounted stent (MicroPort, Shanghai, China), were allowed. If permanent stent deployment was performed, tirofiban (glycoprotein IIb/IIIa inhibitors) was provisionally administered, followed by a loading dose of clopidogrel, and aspirin was immediately administered orally. Daily oral dual antiplatelet therapy was started postprocedure and continued for 3 months. Subsequently, 100 mg of aspirin was prescribed for the rest of the lifetime of the patient (11).

### Outcomes

The primary efficacy endpoint was successful reperfusion, defined as achieving modified thrombolysis in cerebral infarction (mTICI) (12) 2b or 3, in AIS patients assessed by an independent angiography core laboratory. All of the images were read by two experienced neuroradiologists, with consensus required in cases of discrepancy. First-pass effect and modified first-pass effect were also compared. First-pass effect was defined as achieving complete recanalization (mTICI 3) with a single thrombectomy device pass. Modified first-pass effect was defined as meeting mTICI 3/2b after the first pass (13, 14). Major safety outcomes included symptomatic intracranial hemorrhage (sICH) within 24 ± 6 h and all-cause mortality within 90 days, which were assessed by an independent clinical events committee. An sICH was defined as any ICH identified by CT scan combined with a four-point increase in NIHSS or death. The secondary endpoints were time from groin puncture to reperfusion, NIHSS at 24 h and at 7 days, favorable clinical outcomes (defined as an mRS of 0–2), and median mRS score at 90 days. The mRS score at 90 days was done by outpatient follow-up or telephone interview.

### Statistical Analysis

The primary study hypothesis was that the rate of successful reperfusion in the Tonbridge group would be non-inferior to the rate in the Solitaire group. According to results previously reported in the literature, both devices were assumed to have a successful reperfusion rate of 90%. A clinically relevant non-inferiority margin of 12% was chosen as the acceptable difference between groups simultaneously, under a one-sided significance level alpha of 2.5% and an estimated 10% withdrawal or loss to follow-up rate. Under these conditions, randomizing a total of 220 patients would provide 80% power to demonstrate non-inferiority of the Tonbridge stent to that of the Solitaire FR.

Baseline data are presented as descriptive statistics according to treatment assignment, as appropriate. All statistical analyses followed the intention-to-treat principle. Continuous variables are presented as mean ± SD and categorical variables are presented as either counts or percentages. Normally distributed continuous variables were compared using Student's *t*-test. Categorical variables were compared using a chi-squared test or Fisher's exact test. For the primary endpoint, two-sided 95% confidence intervals (CIs) of differences in the rate of successful reperfusion between the groups were estimated by a Cochran–Mantel–Haenszel chi-squared test with adjusting center. The non-inferiority test was based on an asymptotic *Z* test. All the analyses were performed assuming a significance level of two-sided 0.05, using SAS software, version 9.4 (SAS Institute, Cary, NC, USA).

## Results

**Figure 2:**
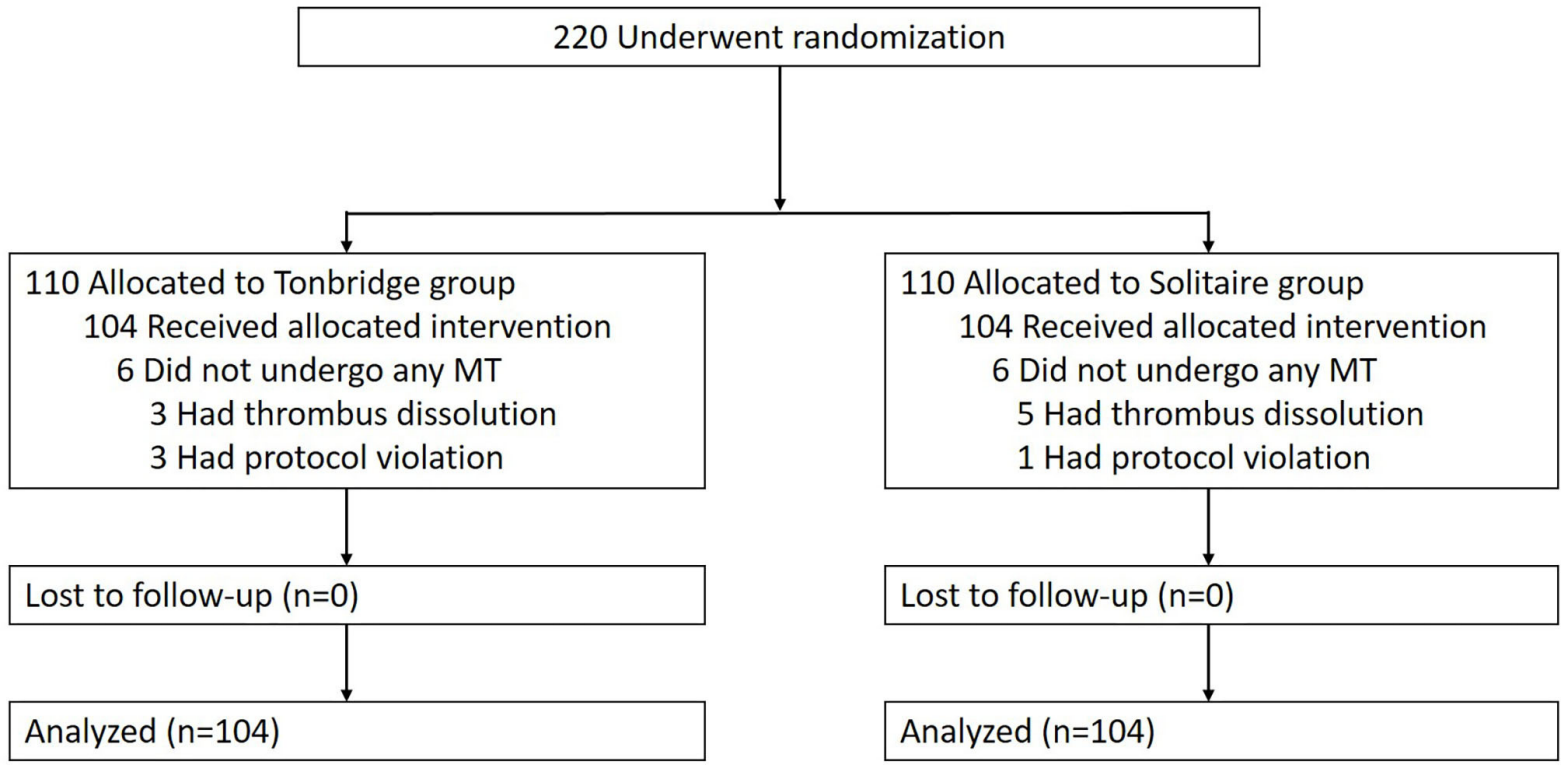
Randomization and treatment of the patients.

Between August 3, 2017, and August 27, 2018, a total of 220 patients were enrolled in the trial (Figure 2). Eight patients had thrombus dissolution before the devices reached the target vessels, and four had protocol violations. A total of 104 patients were treated with the Tonbridge stent, while 104 underwent thrombectomy with the Solitaire FR. No patients crossed over or were lost to follow-up. The baseline characteristics of the patients are detailed in Table 1. The patient median age was 62 years in the Tonbridge group and 61 years in the Solitaire group. There were 52 target vessels in the ICA cases, 133 in the MCA M1 cases, 21 in the MCA M2 cases, and 2 in cases of ACA A2. There were no differences in baseline characteristics observed between the two groups.

**Table 1:** Baseline characteristics of the 208 patients.

**Characteristic**	**Tonbridge group** ** (*n* = 104)**	**Solitaire group** ** (*n* = 104)**	***P***
Age—years, median (IQR)	63.98 (53.96–71.39)	66.50 (55.82–72.76)	0.910
Male sex, no. (%)	62 (59.6%)	61 (58.7%)	0.888
BMI, mean	24.1 ± 3.3	23.8 ± 3.8	0.631
Prestroke mRS 1, no. (%)	10 (9.6%)	14 (13.5%)	0.385
NIHSS—median (IQR)	15 (12–19)	16 (13–19)	0.431
ASPECTS—median (IQR)	9 (8–10)	9 (8–10)	0.580
Systolic blood pressure at hospital arrival—mmHg, median (IQR)	143 (129–160)	143 (127–153)	0.416
Glucose level at hospital arrival—mmol/L, median (IQR)	6.9 (6.0–8.5)	6.8 (6.1–7.8)	0.130
Time from stroke onset to groin puncture—min, median (IQR)	256 (193–315)	248 (187–306)	0.462
**Intracranial arterial occlusion, no. (%)**			
ICA MCA M1 segment MCA M2 segment ACA A1 segment ACA A2 segment	21 (20.2%) 71 (68.3%) 11 (10.6%) 0 (0%) 1 (1.0%)	31 (29.8%) 62 (59.6%) 10 (9.6%) 0 (0.0%) 1 (1.0%)	0.461
**Preprocedure mTICI, no. (%)**			
0 1 2a 2b 3	90 (86.5%) 10 (9.6%) 4 (3.8%) 0 (0%) 0 (0%)	97 (93.3%) 6 (5.8%) 1 (1.0%) 0 (0%) 0 (0%)	0.196

*BMI, body mass index; IQR, interquartile range; mRS, modified Rankin Scale; NIHSS, National Institutes of Health Stroke Scale; ASPECTS, Alberta Stroke Program Early CT score; ICA, internal carotid artery; MCA, middle cerebral artery; ACA, anterior cerebral artery; mTICI, modified thrombolysis in cerebral infarction score*.

Procedural results and outcomes are shown in Table 2. In 37 patients (18 in the Tonbridge group and 19 in the Solitaire group), bridging intravenous fibrinolysis was started before thrombectomy. There were no differences in bridging therapy between the two groups. No balloon guide catheters were used in either arm. In all 104 patients in the Tonbridge group, the Tonbridge was used as the single retriever with no rescuing needed using other thrombectomy devices. The use of a retriever in conjunction with aspiration was similar between the two groups. There were no significant differences observed in the median number of passes needed with the assigned study device between the groups. First-pass effect was achieved in 39/208 patients, with no significant differences noted between the Tonbridge group and the Solitaire group (18.3 vs. 19.2%, respectively). There was a similar result in the modified first-pass effect. Angioplasty with balloon and/or stent was performed as a remedial measure to maintain a stable flow in 26 patients in the Tonbridge group and 16 patients in the Solitaire group (*p* = 0.084). Before angioplasty, 90 patients (86.5%) in the Tonbridge group and 85 (81.7%) in the Solitaire group reached successful reperfusion (*p* = 0.343). Median time from groin puncture to successful reperfusion was similar between treatment groups. Finally, more patients in the Tonbridge group than those in the Solitaire group achieved successful reperfusion (92.3 vs. 84.6%, 95% CI of difference value 0.9%−16.7%, *p* < 0.0001).

**Table 2:** Procedural and outcomes data.

	**Tonbridge group** ** (*n* = 104)**	**Solitaire group** ** (*n* = 104)**	***P***
Bridging intravenous fibrinolysis, no. (%)	18 (17.3%)	19 (18.3%)	0.856
Retriever in conjunction with aspiration, no. (%)	4 (3.8%)	7 (6.7%)	0.353
Number of passes by retriever, median	1.5	1	0.641
Balloon and/or stent angioplasty, no. (%)	26 (25.0%)	16 (15.4%)	0.084
**Primary outcome**:Final successful reperfusion, no. (%)	96 (92.3%)	88 (84.6%)	<0.0001^a^
mTICI 2b/3 with a first pass, no. (%)	50 (48.1%)	46 (44.2%)	0.578^b^
mTICI 3 with a first pass, no. (%)	19 (18.3%)	20 (19.2%)	0.859^b^
mTICI 2b/3 before angioplasty, no. (%)	90 (86.5%)	85 (81.7%)	0.343^b^
**Secondary outcomes**: Time from groin puncture to successful reperfusion—min, median (IQR) NIHSS at 24 h, median (IQR)^c^ NIHSS at 7 days, median (IQR)^d^ mRS 0–2 at 90 days, no. (%) mRS at 90 days, median (IQR)	67.5 (46.5–95.5) 10 (4–15) 6 (2–13) 61 (58.7%) 2 (1–4)	67 (44.0–99.0) 8 (4–16) 4 (1–13) 64 (61.5%) 2 (1–4)	0.970 0.993 0.777 0.671 1.000
**Postprocedure mTICI, no. (%)**			
0 1 2a 2b 3	1 (1.0%) 3 (2.9%) 4 (3.8%) 64 (61.5%) 32 (30.8%)	4 (3.8%) 3 (2.9%) 9 (8.7%) 58 (55.8%) 30 (28.8%)	0.3138
**Major safety outcomes:** Symptomatic intracranial hemorrhage within 24 h, no. (%)^e^ Death within 90 days, no. (%)	2 (1.9%) 14 (13.5%)	5 (5.0%) 17 (16.3%)	0.238 0.559

*mTICI, the modified thrombolysis in cerebral infarction score; NIHSS, National Institutes of Health Stroke Scale; mRS, modified Rankin Scale.*

a *The p-value was for noninferiority and calculated by the CMH chi-square test.*

b *The p-value was for difference and calculated by the chi-square test.*

c *Data were missing for one patient in the Tonbridge group and four patients in the Solitaire group.*

d *Data were missing for seven patients in the Tonbridge group and 11 patients in the Solitaire group.*

e *Data were missing for one patient in the Tonbridge group and three patients in the Solitaire group*.

Regarding other group comparisons, differences in sICH within 24 h (1.9% in the Tonbridge group and 5.0% in the Solitaire group) and all-cause mortality within 90 days (13.5% in the Tonbridge group and 16.3% in the Solitaire group) were not significant. We also noted no significant differences between groups in terms of NIHSS at 24 h and NIHSS at 7 days. Rates of favorable outcome (mRS 0–2) at 90 days were 58.7% in the Tonbridge group and 61.5% in the Solitaire group (*p* = 0.671).

## Discussion

Our randomized controlled trial demonstrated that the Tonbridge stent had a comparable rate of successful reperfusion when compared with the Solitaire FR in a clinical setting for the treatment of AIS due to large vessel occlusion in the anterior circulation. In terms of safety endpoints, sICH within 24 ± 6 h and all-cause mortality within 90 days were comparable for both devices. Clinical outcomes within 7 days and at 90 days follow-up were comparable between the two groups.

Although several approved thrombectomy devices are currently available, improvements designed to increase the effectiveness of reperfusion have continued. The Tonbridge is a novel stent-based clot retriever with a hybrid closed and partially open cell design, which forms a longitudinal spiral opening along its tubular surface. It was designed to allow increases in stent radial force strength and the tensile breaking force of the connection point between the stent and its push wire. In *in vitro* tests, the radial force and flexibility of the Tonbridge stent were superior to those of the Solitaire FR (6). These improvements may have the following potential advantages: the promise of tighter clot engagement and reduced chance of clot fall-off during retrieval; they make the device better adapted to curved vessel lumen; and they prevent connection site break-off during repeated retriever manipulations. Animal experiments revealed a slightly higher rate of recanalization (100 vs. 88.9%), but without statistical significance (6). To determine the value of this novel device in the treatment of AIS, this clinical comparative study was essential.

Our study is one of the few prospective randomized controlled trials that focuses on the efficacy and safety of a new stent-like device. Trials of thrombectomy devices have usually been single-arm studies whose aim was to show the safety of a device for regulatory approval purposes (15–23). In these studies, the data from a single group were usually compared with the data from previous studies of other devices, such as the Solitaire FR or the Trevo. These two devices have been evaluated in numerous trials and prospective registries (16, 18). However, direct comparison of prognoses between single-arm studies and previous registries can produce uncontrolled effects from inhomogeneous baselines and different endpoints. In our study, the two groups had well-balanced baseline characteristics because of the prospective randomization design. To minimize bias, the assessments of key endpoints, including postprocedural mTICI and sICH, were evaluated by an independent core laboratory and clinical events committee.

The Tonbridge stent achieved a similar rate of successful reperfusion as the Solitaire FR before angioplasty. We also compared first-pass effect and modified first-pass effect between the two groups. These indicators were first described by Zaidat et al. (13) and have subsequently been shown to be independent factors of favorable outcomes (14, 24, 25). In this trial, a first-pass effect was achieved in one-fifth of all patients, which was slightly lower than that reported by Zaidat as well as a recently published systematic review (13, 26). Potential reasons may be a lack of the use of balloon guide catheters, which has been proven to contribute to a greater first-pass effect. Our trial indicates a similar rate of modified first-pass effect to that of previous studies (13, 14, 24–26).

The fact that acute arterial occlusions due to intracranial atherosclerotic disease (ICAD) are more prevalent in Eastern populations was an issue. Recent studies have shown that ICAD-related occlusion accounts for 12%−30% of all large vessel occlusion etiologies in Asia (27–30). However, while the thrombectomy devices could achieve initial reperfusion quickly, reocclusion is often encountered due to subsequent platelet aggregation at the lesion site or elastic recoil. In our study, about one-fifth of patients received angioplasty with a balloon and/or stent in order to maintain successful reperfusion. In line with a study published by Yoon et al., these patients achieved a higher recanalization rate (31). The final successful reperfusion rate we report is higher than those reported in the MR CLEAN, REVASCAT, and ESCAPE trials (1–3) and comparable with those seen in the SWIFT, PRIME, and EXTEND-IA trials (4).

Another concern about the safety of the device was whether the high radial force of the Tonbridge stent would lead to vessel wall injury or hemorrhage. Gascou et al. estimated that >10% of perioperative complications are associated with thrombectomy devices (32), including arterial perforation (0.9–4.9%), subarachnoid hemorrhage (0.6–4.9%), arterial dissection (0.6–3.9%), and vasospasm (3.9–23%) (27, 33). It is well-known that high radial force is associated with higher rates of clot removal (34). However, high radial force is also associated with endothelial vessel wall injury (35, 36). Therefore, minimizing radial force may reduce the incidence of vessel wall injury complications while still maintaining optimal clot retrieval rates. In an *in vitro* test, the radial force of the Tonbridge stent was noted to be slightly higher than that of the Solitaire FR. In the present clinical trial, we did not note any significant differences between the two groups in terms of symptomatic intracranial hemorrhage, including subarachnoid hemorrhage. These results indicate that the Tonbridge stent has the potential to improve the rate of reperfusion without increasing vessel wall injury complications.

Our study was limited in many aspects by the restrictive nature of a randomized controlled trial design, such as strict inclusion and exclusion criteria, center selection, and operator experience. The image core lab evaluated the DSA only without CTA or MRA, and the analysis item was very limited. We acknowledged that these were all the limitations. Postmarketing clinical registries allow for more inclusive criteria by including a range of clinical sites, operators, and varying patient populations, which will provide valuable information on the generalizability and reproducibility of our results, and allow additional opportunities to explore clinical hypotheses for patients treated outside of this trial.

## Conclusions

In this randomized clinical trial, the Tonbridge stent was non-inferior to the Solitaire FR in the treatment of large vessel occlusion stroke within 6 h of symptom onset.

## Data Availability Statement

The raw data supporting the conclusions of this article will be made available by the authors, without undue reservation.

## Ethics Statement

The studies involving human participants were reviewed and approved by Shanghai Changhai Hospital Ethics Committee. The patients/participants provided their written informed consent to participate in this study.

## Author Contributions

YongxZ and WH: manuscript writing and revision. JL and PY: conception and design of the work. All authors: data collection. All authors read and approved the final manuscript.

## Conflict of Interest

QZ was employed by the company Zhuhai Ton-Bridge Medical Tech. Co., Ltd. The remaining authors declare that the research was conducted in the absence of any commercial or financial relationships that could be construed as a potential conflict of interest. The authors declare that this study received funding from Zhuhai Ton-Bridge Medical Tech. Co., Ltd. The funder had the following involvement in the study: study design.

## Publisher's Note

All claims expressed in this article are solely those of the authors and do not necessarily represent those of their affiliated organizations, or those of the publisher, the editors and the reviewers. Any product that may be evaluated in this article, or claim that may be made by its manufacturer, is not guaranteed or endorsed by the publisher.
